# Amyloid fibril composition type is consistent over time in patients with Val30Met (*p*.*Val50Met*) transthyretin amyloidosis

**DOI:** 10.1371/journal.pone.0266092

**Published:** 2022-03-31

**Authors:** Intissar Anan, Ole B. Suhr, Katarzyna Liszewska, Jorge Mejia Baranda, Björn Pilebro, Jonas Wixner, Elisabet Ihse

**Affiliations:** 1 Department of Public Health and Clinical Medicine, Umeå University, Umeå, Sweden; 2 Wallenberg Centre for Molecular Medicine, Umeå University, Umeå, Sweden; 3 Department of Medicine, Piteå Hospital, Piteå, Sweden; 4 Department of Immunology, Genetics and Pathology, Uppsala University, Uppsala, Sweden; Heart and Diabetes Center NRW, UNiversity Hospital of the Ruhr-University Bochum, GERMANY

## Abstract

**Background:**

We have previously shown that transthyretin (TTR) amyloidosis patients have amyloid fibrils of either of two compositions; type A fibrils consisting of large amounts of C-terminal TTR fragments in addition to full-length TTR, or type B fibrils consisting of only full-length TTR. Since type A fibrils are associated with an older age in ATTRVal30Met (*p*.*Val50Met*) amyloidosis patients, it has been discussed if the TTR fragments are derived from degradation of the amyloid deposits as the patients are aging. The present study aimed to investigate if the fibril composition type changes over time, especially if type B fibrils can shift to type A fibrils as the disease progresses.

**Material and methods:**

Abdominal adipose tissue biopsies from 29 Swedish ATTRVal30Met amyloidosis patients were investigated. The fibril type in the patients´ initial biopsy taken for diagnostic purposes was compared to a biopsy taken several years later (ranging between 2 and 13 years). The fibril composition type was determined by western blot.

**Results:**

All 29 patients had the same fibril composition type in both the initial and the follow-up biopsy (8 type A and 21 type B). Even patients with a disease duration of more than 12 years and an age over 75 years at the time of the follow-up biopsy had type B fibrils in both biopsies.

**Discussion:**

The result clearly shows that the amyloid fibril composition containing large amounts of C-terminal fragments (fibril type A) is a consequence of other factors than a slow degradation process occurring over time.

## Introduction

Systemic amyloidoses are a group of fatal diseases, in which plasma proteins form aggregates with a specific fibrillar conformation called amyloid. As the aggregating protein is a plasma protein, which reaches most organs, amyloid deposits can form throughout the body. However, for largely unknown reasons, the deposits are not homogenously distributed in the body, but certain tissue types or organs are more affected than others. Which tissue that is most heavily affected depends somewhat on the precursor protein (i.e. type of amyloidosis), but large differences in deposition pattern exist also between patients suffering from the same type of amyloidosis [1].

One protein that can cause systemic amyloidosis is transthyretin (TTR), a homotetrameric protein acting as a transport protein in plasma, that may have other less investigated functions in other parts of the body as well [2]. The disease, termed ATTR amyloidosis, can either be caused by the wild-type protein [3] or be facilitated by a mutation in the TTR gene [4]. New mutations are still being reported, and around 200 mutations have been found, which results in more than 180 different variants of the secreted protein (see Human Gene Mutation database, accessible at www.hgmd.cf.ac.uk). Most of these variants have been found to be amyloidogenic [4]. As with other systemic amyloidoses, the deposition pattern of the amyloid and the resulting symptoms differ between patients, but cardiac and neurological manifestations are the most common. In addition, deposition in other sites like kidneys, eyes and CNS can cause tissue damage as well [5]. The disease phenotype depends to some extent on the genotype [4], but variations in disease phenotype are seen even among patients carrying the same mutation [6–8].

The Val30Met mutation in the TTR gene is the most studied variant. It is well established that ATTR amyloidosis patients with this mutation can roughly be divided into two phenotypic groups [6, 8, 9]. Some patients display significant peripheral autonomic neurological disturbances as well as cardiac arrhythmias caused by amyloid deposits predominantly around the conduction system, while other patients present with less neuropathy but instead develop amyloid cardiomyopathy, caused by massive amyloid deposits throughout the myocardium, leading to heart failure [6, 7]. The first-mentioned group of patients tends to have an earlier onset of disease than the latter group. In lack of an understanding of the mechanisms behind the phenomenon of the two phenotype groups, a cut-off point to separate them was decided by using the age of onset of disease (usually 50 years of age but an age of 40 years is sometimes used) and patients are often referred to as “early” or “late onset” cases [6, 10].

We have earlier described that the fibril composition in ATTR amyloidosis can be of two distinct types. In some patients, the amyloid deposits consist of a mixture of full-length TTR and large amounts of C-terminal TTR fragments (denominated fibril type A), while in other patients, the deposits consist of only full-length TTR (fibril type B) [11, 12]. We have shown that the fibril conformation type is strongly correlated to the two clinical phenotypes among ATTRVal30Met amyloidosis patients, thereby offering a plausible explanation for the presence of the two groups [12]. For example, cardiac enlargement and an older age of onset are seen among patients with type A fibrils, while no or little cardiac enlargement and a younger age of onset is seen among patients with type B fibrils [12]. In addition, amyloid fibril composition probably has an impact on outcome after liver transplantation for ATTRv amyloidosis, since late onset ATTRV30M patients had an inferior survival to that of early onset patients [13], and late onset of ATTRV30M amyloidosis also appears to decrease the efficacy of Tafamidis treatment [14]. Considering the possible impact of amyloid fibril type on the outcome of treatment modalities for ATTR amyloidosis, the consistency over time of ATTR fibril type are of clinical importance.

We hypothesize that the two fibril composition types are similar to prion “strains”, where fibrillar conformation variations cause differences in deposition patterns, and where the particular fibrillar conformation persist when the fibrils grow and spread [15, 16]. However, the fact that there is a correlation between older age and type A fibrils (i.e. presence of large amounts of TTR fragments in the amyloid fibrils), raises the question if the fragments are formed over time as a consequence of degradation of the deposits, thereby leading to a shift from type B to type A amyloid fibrils.

To answer if the fibril composition type can change with time, we compared the amyloid fibril type between initial and follow-up biopsies from ATTRVal30Met amyloidosis patients.

## Material and methods

### Patients and tissue specimens

ATTRVal30Met amyloidosis patients treated at the Amyloidosis Center at Umeå University Hospital and the FAP-team at Piteå Hospital, and whose amyloid fibril composition type had been determined at the diagnostic unit for amyloidosis at Uppsala University Hospital, were included in this study. All patients were heterozygous for the Val30Met mutation, and compound heterozygosity was not present. Their medical journals were reviewed for age at onset of disease, age at biopsy procedures, organ transplantation, clinical presentation and medical treatments.

**Fig 1:**
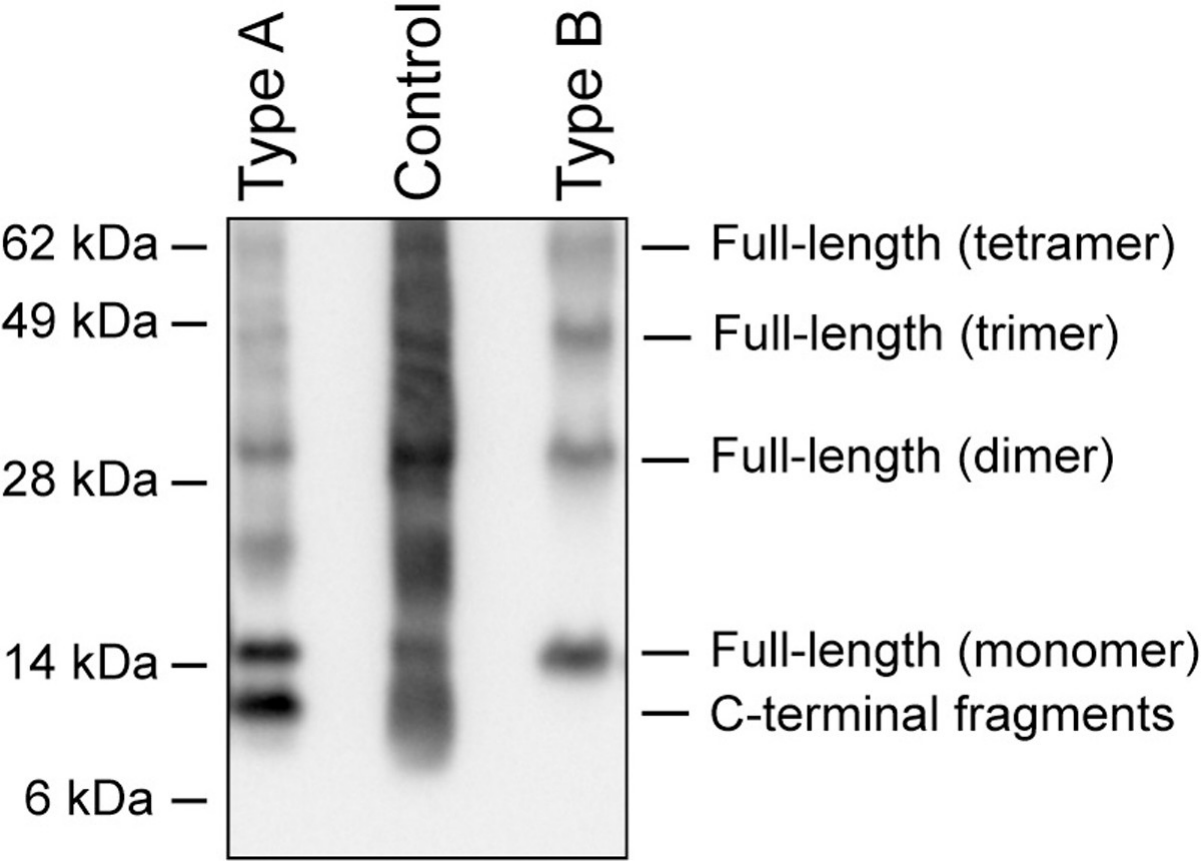
Typical examples of fibril composition type A and type B, visualized by western blot. A monoclonal antibody directed against TTR50-127 was used, which detects full-length TTR as well as the C-terminal fragments found in type A fibrils. Control = Cardiac tissue containing ATTR amyloid fibrils of type A.

Unfixed abdominal adipose tissue obtained through a punch biopsy were used to diagnose the patients and to determine the ATTR amyloid fibril composition type. Diagnosis of amyloidosis was done by Congo red staining and polarization microscopy, while determination of type of amyloidosis (to ATTR) and the fibril conformation type (to A or B) was performed by western blot analysis (Fig 1).

At the time of this study, the fibril type had been determined for 209 ATTR amyloidosis patients, who were treated at the Amyloidosis center in Umeå and the FAP-team in Piteå. Most of these patients carried the TTRVal30Met mutation.

For the first part of this study, the medical records were reviewed to identify patients carrying the TTRVal30Met mutation, who already had at least two fibril type determined biopsies, with more than one year in between the procedures. Thirteen patients fulfilled these criteria (Table 1).

**Table 1:** Patients whose fibril type already had been determined in two biopsies.

Patient number	Sex	Fibril type at first / last biopsy	Duration between biopsies (years)	Age at onset of disease (years)	Age at first / last biopsy (years)	Phenotype at first/last biopsy	Transplantation (organ / age (years))	Medical treatment for ATTR amyloidosis
1	F	B / B	6.2	25	29 / 35	PN (1), GI / PN (1), GI	Liver / 30	None
2	M	B / B	3.5	36	38 / 41	PN (1) / PN (1)	Liver / 39	None
3	F	B / B	2.6	42	49 / 52	GI / GI	Liver / 49	None
4	M	B / B	6.2	61	59 / 65	Eye / Eye, Kidney	Liver + kidney / 66	None
5	M	B / B	3.2	58	64 / 67	PN (1), CM, GI / PN (1), CM, GI	Liver / 62	None
6	M	B / B	3.9	63	65 / 69	PN (1), CM, GI/ PN (1), CM	Liver / 65	None
7	M	B / B	2.2	66	78 / 80	PN (1), CM / PN (3), CM	No	None
**Mean ± SD**			4.0 ± 1.6	50±16	55 ± 17 / 58 ± 16			
8	M	A / A	3.0	50	52 / 55	PN (1) / PN 1), CM	Liver / 53	None
9	M	A / A	2.1	55	58 / 60	PN (1), CM / PN (1), CM, GI	Liver / 55	Diflunisal
10	M	A / A	3.3	59	59 / 62	PN (1), CM, GI / PN (1), CM, GI	Liver / 60	Diflunisal
11	M	A / A	3.7	61	61 / 65	PN (1), GI / PN (1), CM	Liver / 62	None
12	M	A / A	2.7	59	63 / 65	PN (1), CM / PN (1), CM	Liver / 64	None
13	M	A / A	2.2	63	70 / 72	PN (2), CM / PN(NA), CM, GI	No	None
**Mean ± SD**			2.8 ± 0.6	58±4.7	61 ± 6.0 / 63 ± 5.7			

PN = Polyneuropathy, within brackets (stage (1–3), where 1 denotes walking without support, 2 walking with support, and 3 bedridden or bound to a wheelchair); GI = Gastrointestinal complications; Eye = Vitreous opacities, glaucoma; Kidney = Kidney failure; CM = Cardiomyopathy. NA = data not available.

For the second part of the study, the medical records were reviewed to identify patients who carried the TTRVal30Met mutation and who had a fibril type determined biopsy taken at least 5 years earlier. The criteria were met by 119 patients, of whom 56 individuals showed type A fibrils and 63 individuals showed type B fibrils. Patients who were no longer alive or not deemed in good enough health for a new biopsy procedure were excluded. The resulting 26 patients were asked to volunteer for a long-term follow-up biopsy. Sixteen patients consented and went through with the biopsy procedure (Table 2).

**Table 2:** Patients from whom a long-term follow-up biopsy was collected for this study.

Patient number	Sex	Fibril type at initial / follow-up biopsy	Duration between biopsies (years)	Age at onset of disease (years)	Age at initial / follow-up biopsy (years)	Phenotype at initial/follow-up biopsy	Transplantation (organ / age (years))	Medical treatment for ATTR amyloidosis
14	M	B / B	6.0	28	31 / 37	PN (1) / PN (1)	Liver, /31	None
15	M	B / B	7.0	32	34 / 41	PN (1), GI / PN (NA), GI	Liver / 34	Diflunisal
16	M	B / B	10.2	35	35 / 45	PN (1) / PN (1)	Liver / 36	None
17	M	B / B	11.2	39	40 / 52	PN (1) / PN (1)	Liver / 41	None
18	F	B / B	12.9	39	41 / 54	PN (1), GI / PN (1), GI	Liver / 41	None
19	M	B / B	10.9	38	43 / 54	PN (1), GI / PN (1), GI	Liver / 40	None
20	M	B / B	9.3	47	49 / 58	PN (1) / PN (1)	Liver / 49	None
21	M	B / B	8.5	51	52 / 61	PN (1) / PN (1)	Liver, /53	None
22	F	B / B	9.8	50	55 / 65	PN (1), GI / PN (2), GI	Liver, /56	None
23	M	B / B	12.1	54	55 / 67	PN (1)/ PN (1)	Liver / 56	None
24	M	B / B	8.6	52	56 / 64	PN (1), GI / PN (1), CM, GI	Liver / 57	None
25	M	B / B	11.2	61	64 / 76	PN (1), CM / PN (NA), CM	Liver / 62	None
26	M	B / B	12.2	53	65 / 77	PN (1), CM, GI / PN (2), CM, GI	Liver / 59	None
27	M	B / B	6.3	67	68 / 75	PN (1), GI / PN (1), GI	No	Patisiran
**Mean ± SD**			9.8 ± 2.2	46±11	49 ± 12 / 59 ± 13			
28	M	A / A	11.7	60	60 / 71	PN (1), CM / PN (1)	Liver + heart / 61	Diflunisal
29	F	A / A	7.1	65	66 / 73	PN (1), CM / PN (1), CM	No	Patisiran
**Mean ± SD**			9.4 ± 3.3	63±3.5	63 ± 4.2 / 72 ± 1.4			

PN = Polyneuropathy, within brackets (stage (1–3), where 1 denotes walking without support, 2 walking with support, and 3 bedridden or bound to a wheelchair); GI = Gastrointestinal complications; CM = Cardiomyopathy; NA = data not available.

All but four patients had been liver transplanted as a result of their diagnosis. Patient 4 had undergone a combined liver and kidney transplantation, and patient 28 had undergone a sequential heart and liver transplantation.

The study was approved by the Regional Ethics Board in Umeå, Sweden (reference number 2017–447-31M) and written informed consent was obtained from all participants.

### Congo Red staining

Congo Red staining was performed to determine if amyloid was present in the biopsies. Non-fixed abdominal adipose tissue biopsies were cut into small pieces and squashed between two microscope slides as previously described in detail [17]. The slides were dried on a heating plate for 10 minutes, followed by defatting of the tissue in acetone and staining with Congo red [17]. The samples were thereafter examined with polarization microscopy to determine if amyloid was present. Biopsies with detected amyloid deposits were further analyzed by SDS-PAGE and Western Blot Analysis.

### SDS-PAGE and Western Blot analysis

Non-fixed abdominal adipose tissue biopsies were prepared for gel electrophoresis as described in detail in [12]. In short, the biopsy tissue was washed in an isotonic NaCl solution, then incubated in an ammonium sulfate solution for lysis of erythrocytes, which was followed by defatting in acetone. Thereafter, the samples were left to air-dry.

A sample buffer containing 4% sodium dodecyl sulfate, 20 mM dithiothreitol and 40 mM iodoacetamide was added to the dried tissue and the samples were heated at 96°C before loaded onto a 4–12% polyacrylamide gel (NuPAGE, Thermo Fisher Scientific, Waltham, MA, USA). After gel electrophoresis, the proteins were transferred to a nitrocellulose membrane. The membrane was blocked in a 5% milk TBS-Tween solution, and incubated with either a polyclonal rabbit antiserum or a monoclonal mouse antibody against TTR50-127 (described in [18, 19]), followed by an anti-rabbit or anti-mouse antibody coupled to horse-radish peroxidase (HRP) (Agilent, Santa Clara, CA, USA). The reaction was developed using enhanced chemiluminescence (ECL) (Millipore, Burlington, MA, USA) and captured with a CCD-camera (Bio-Rad Laboratories, Hercules, CA, USA).

## Results

### Patients who had already undergone more than one biopsy

**Fig 2:**
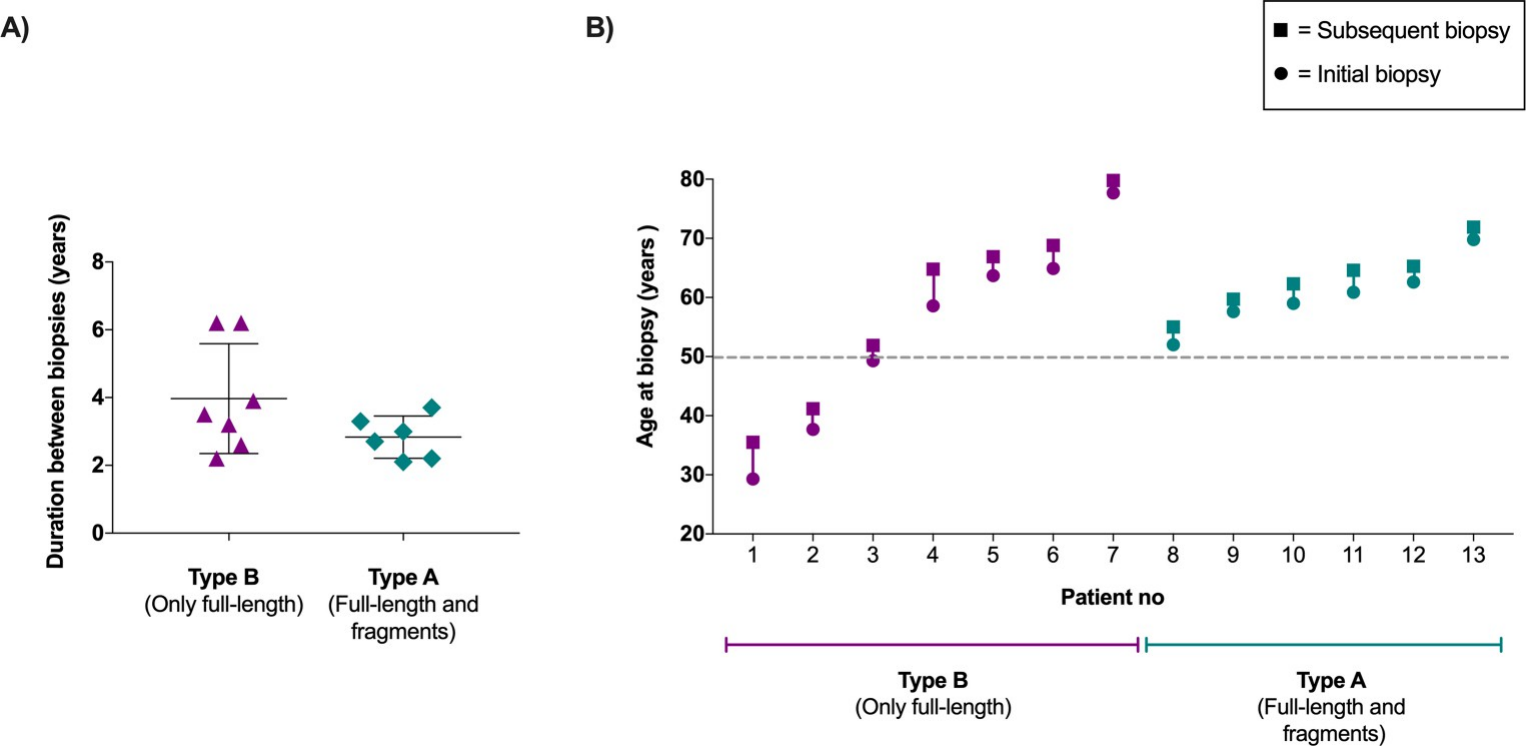
Duration and age, for patients whose fibril type already had been determined in two biopsies. (A) Duration between the initial and subsequent biopsy, grouped according to fibril type. Horizontal lines represents the mean value ± standard deviation. (B) Age at initial and subsequent biopsy. The grey dashed line represents the age at disease onset (50 years old) which most often is used to determine a patient as “early or late onset”.

To investigate if the amyloid fibril type may change over time, we reviewed the medical records for ATTRVal30Met amyloidosis patients from whom more than one biopsy had been taken, and the fibril type had been successfully determined for at least two of those biopsies. In all 13 patients who fulfilled the inclusion criteria, the same fibril type was present in both the initial and the sequential biopsy, i.e. the fibril type did not change over time (Table 1, Fig 2). Six patients had fibrils of type A, and seven patients had fibrils of type B. However, the duration between the first and last biopsy for each of these patients was relatively short, ranging between 2 to 4 years for type A patients and 2 to 6 years for type B patients (Fig 2).

### Patients recruited for a long-term follow-up biopsy

To study if the fibril type may change over a longer time span, patients whose fibril type had been determined, were recruited for a voluntary long-term (> 5 years) follow-up biopsy.

**Fig 3:**
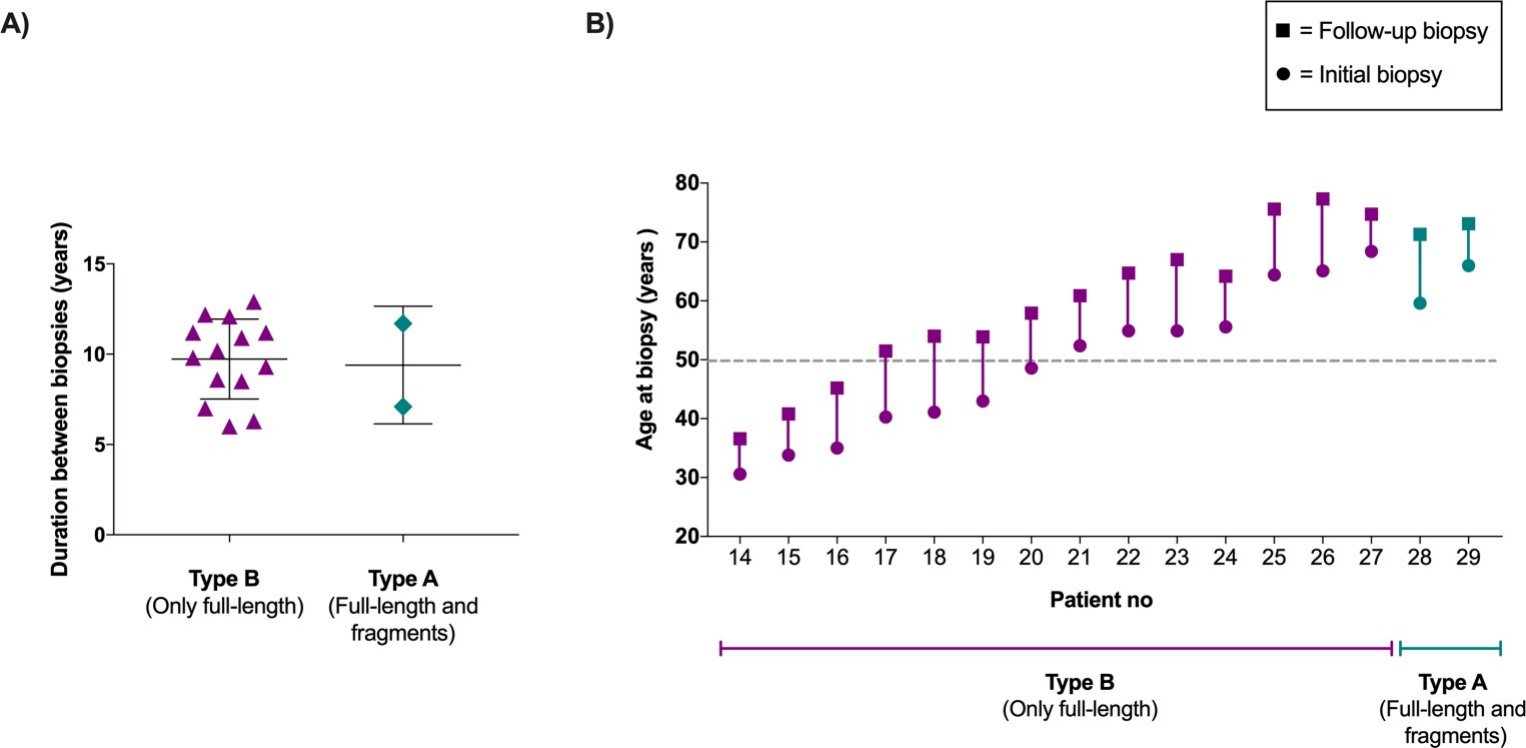
Duration and age, for patients from whom a long-term biopsy was collected for this study. (A) Duration between the initial and follow-up biopsy grouped according to fibril type. Horizontal lines represent the mean value ± standard deviation. (B) Age at the initial and the long-term follow-up biopsy. The grey dashed line represents the age at disease onset (50 years old) which most often is used to determine a patient as “early or late onset”.

Sixteen patients underwent the follow-up biopsy, and in all patients, the same fibril type was seen in the follow-up biopsy as in the initial biopsy (Table 2, Fig 3). The vast majority of these patients had amyloid with type B fibrils, and only two had type A fibrils. The duration between the initial and follow-up biopsies ranged from 6 to 13 years among type B patients and 7 to 12 years among type A patients (Fig 3A). The age at the initial biopsy ranged from 31 to 68 years among type B patients and from 60 to 66 years among type A patients, while at the follow-up biopsy the age ranged from 37 to 77 years among type B patients and from 71 to 73 years among type A patients (Fig 3B). Interestingly, some type B patients (patient no 25–27) had a comparable age at the initial and follow-up biopsies to the two type A patients (patient no 28–29) (Table 2 and Fig 3B), but their amyloid fibrils were of type B in both biopsies.

## Discussion

The present study clearly shows that an amyloid fibril composition of only full-length TTR (fibril type B) is consistent over time in ATTRVal30Met amyloidosis patients and does not develop into fibrils with large amounts of fragmented TTR (fibril type A). Hence, the presence of ATTR amyloid fibrils consisting of a mixture of full-length TTR and large amounts of TTR fragments in mainly elderly ATTR Val30Met amyloidosis patients cannot be explained by differences in disease duration and the “age” of the amyloid deposits. Instead, it seems plausible that the two fibril conformation types stems from two distinct fibril formation mechanisms.

Even though the most important aspect of this study was to investigate if type B fibrils could develop into type A fibrils over time, we also wanted to study follow-up biopsies on patients whom showed fibril type A deposits at the initial biopsy. Unfortunately, we had difficulties in recruiting such patients for a long-term follow-up biopsy, since most were either deceased or their disease was too advanced for them to undergo a second biopsy.

All but two of the patients from whom a long-term follow-up biopsy were taken had undergone liver transplantation. Differences in survival rate after liver transplantation between patients with the two different fibril types have not been investigated, but it is known that “late onset” Val30Met patients have a drastically lowered survival compared to “early onset” Val30Met patients after liver transplantation [13]. In addition, when type A and B patients of almost comparable mean ages were studied, patients with type A fibrils developed cardiac function impairment after liver transplantation, whereas such deterioration was not found among type B patients [20]. In light of this, it is noteworthy that the only type A patient with a relatively long duration between the two biopsy procedures (patient no 28), had undergone both a heart and liver transplantation.

It should be mentioned that this study is performed on amyloid in only one type of tissue (abdominal adipose tissue) and in one TTR mutation (Val30Met). We cannot exclude that changes in amyloid fibril conformation can occur over time in other tissue types or in other mutations. However, it seems reasonable to believe that such changes do not occur, as we have previously shown that the fibril type is intra-individually consistent between different tissue types [12, 21]. It is unknown if a similar correlation between fibril type and phenotypic differences as is seen among Val30Met patients occurs in patients with other TTR variants. However, when investigating the fibril composition type in several other mutations than Val30Met [22], we found that regardless of age or cardiac involvement, all but one patient (out of 62) had fibrils of type A. Together with the fact that all wild-type ATTR patients have type A fibrils [11], it seems likely that the fibril type B is rare among ATTR amyloidosis patients and may only occur in a few mutations.

It could be argued that if there indeed are two different mechanisms behind the formation of type A and type B fibrils, both of them could, at least in theory, occur in the same patient. Especially of interest is if the mechanism behind type A fibril formation could start to occur in addition to the type B formation mechanism in a few patients who have type B fibrils as they reach high age, just as the type A mechanism start to occur in those individuals who develop wild-type ATTR amyloidosis as they reach high age (as all wild-type patients present with type A fibrils [11]). Such patients would perhaps develop the typical cardiomyopathy associated with type A fibrils. This hypothetical, and likely rare, scenario should however be differentiated from a scenario where all type A fibrils start out as type B fibrils, as the latter scenario seems very unlikely according to the result of the present study, where no presence of the typical C-terminal fragments was seen in the follow-up biopsies of the type B patients. In this regard, it is also interesting to note that, even though patients with wild-type ATTR amyloidosis always have amyloid fibrils of type A [11], and we know that wild-type TTR is continued to be incorporated into the amyloid after liver transplantation [23], the patients in this study who showed fibril type B before liver transplantation continued to have amyloid consisting of only type B fibrils many years after the procedure. This further indicates that there are either two different fibrillization mechanisms for ATTR amyloidosis which differs between patients, or that seeding is taking place, causing the original structure and organization of the fibrils to be perpetuated even when no mutant TTR is being incorporated.

It is still unclear if the cleavage process that produces the TTR fragments found in type A ATTR fibrils, occurs before or after incorporation of the protein into the fibrils. Even though type A fibrils seem to be the most common fibril composition in ATTR amyloidosis [11, 22], surprisingly little attention has been paid to the presence of these fragments and their possible role in the ATTR fibrillogenesis. However, in recent years, the interest has increased, and a few reports concerning the fragments have been published. Bellotti *et al*. have used an approach where C-terminal fragments are produced by trypsin treatment of native TTR, and have shown that such treatment triggers formation of amyloid aggregates at a physiological pH [24]. In contrast, full-length tetrameric native TTR will not form amyloid fibrils at physiological pH, but requires acidic conditions [25]. These studies suggest that cleavage is the triggering event for type A fibril formation, and that substances aimed at inhibiting the cleavage may be an important way to hinder fibrillation in patients with type A fibrils [26].

Other studies indicate that full-length TTR is incorporated into the fibrils, and that the cleavage occurs thereafter. A cryo-electron microscopy study on ATTRVal30Met *ex-vivo* amyloid fibrils, which had the fibril type A composition [11], showed a structure that is in line with cleavage after incorporation [27]. If that is the case, such cleavage must occur relatively quickly after incorporation, and cannot be delayed by years, according to the result of the present study. In this regard, it is interesting to note that we have earlier reported, that the increase in the proportion of wild-type to variant TTR seen in fibril type A patient after liver transplantation, seems to occur slightly faster among full-length ATTR protein compared to truncated ATTR protein [23]. Ando *et al*. have also presented evidence that C-terminal TTR fragments arise when aggregated, but not native, TTR is added to certain cell types *in vitro* [28].

The abovementioned studies do not necessarily contradict each other, since it is possible that cleavage of TTR can be the triggering event for fibril formation in type A fibrils, while elongation of these fibrils may occur by full-length TTR incorporation into a fibril structure that promotes a subsequent cleavage.

As mentioned, the two fibril conformation types correlate to both clinical symptoms and age of onset. This strongly suggest that the difference in fibril conformation is the underlying mechanism behind the clustering of ATTRVal30Met amyloidosis patients into two phenotypic groups. Therefore, we believe it would be advantageous to use the fibril type as the defining parameter of the groups, instead of an arbitrarily chosen age of onset. Using the fibril type as the defining parameter of the groups has important clinical implications, as not all patients with type B fibrils have an age at onset below 50 years of age. Therefore, the clinical manifestations of so called “late onset” patients cannot automatically be expected to include severe cardiac enlargement.

The discrepancy in defining the two phenotypic groups may not only be a problem in the clinical setting, but also complicates attempts to find the causative mechanism behind the two phenotypes. Most of the studies aimed to solve this question have used the age of onset to define the groups and not the fibril composition [6, 10, 29, 30]. As the “late onset” group will contain not only patients with type A fibrils, but also those with type B fibrils, important factors behind the phenotypic clustering may go undiscovered. In addition, the age used as a cut-off point for late and early onset differs between studies [6, 10, 29, 30]. This lack in consistency of how to define the two phenotypic groups shows that using “age of onset” is not an optimal parameter.

In conclusion, the two ATTR fibril composition types are stable and do not change with time, indicating that there are two different fibrillization mechanisms in ATTR amyloidosis. The role of TTR fragments in ATTR fibrillogenesis and pathogenesis needs to be further investigated, as well as why fragments are present in the amyloid fibrils of some patients but not others. Knowledge emerging from such studies is likely to give insight into possible implications of fibril type in regards to the newly developed medical treatments as well as lead to improved treatment modalities and more personalized treatment regimes in ATTR amyloidosis.

## Supporting information

S1 Raw images (PDF)

## References

[1] Westermark P. Aspects on human amyloid forms and their fibril polypeptides. The FEBS journal. 2005;272(23):5942–9. doi: 10.1111/j.1742-4658.2005.05024.x . 16302959

[2] Buxbaum JN, Reixach N. Transthyretin: the servant of many masters. Cell Mol Life Sci. 2009;66(19):3095–101. doi: 10.1007/s00018-009-0109-0 . 19644733 PMC4820353

[3] Westermark P, Sletten K, Johansson B, Cornwell GG 3rd. Fibril in senile systemic amyloidosis is derived from normal transthyretin. Proceedings of the National Academy of Sciences of the United States of America. 1990;87(7):2843–5. doi: 10.1073/pnas.87.7.2843 ; PubMed Central PMCID: PMC53787. 2320592 PMC53787

[4] Rowczenio DM, Noor I, Gillmore JD, Lachmann HJ, Whelan C, Hawkins PN, et al. Online registry for mutations in hereditary amyloidosis including nomenclature recommendations. Human mutation. 2014;35(9):E2403–12. Epub 2014/07/22. doi: 10.1002/humu.22619 . 25044787

[5] Hou X, Aguilar MI, Small DH. Transthyretin and familial amyloidotic polyneuropathy. Recent progress in understanding the molecular mechanism of neurodegeneration. The FEBS journal. 2007;274(7):1637–50. doi: 10.1111/j.1742-4658.2007.05712.x . 17381508

[6] Koike H, Misu K, Ikeda S, Ando Y, Nakazato M, Ando E, et al. Type I (transthyretin Met30) familial amyloid polyneuropathy in Japan: early- vs late-onset form. Archives of neurology. 2002;59(11):1771–6. doi: 10.1001/archneur.59.11.1771 . 12433265

[7] Koike H, Misu K, Sugiura M, Iijima M, Mori K, Yamamoto M, et al. Pathology of early- vs late-onset TTR Met30 familial amyloid polyneuropathy. Neurology. 2004;63(1):129–38. doi: 10.1212/01.wnl.0000132966.36437.12 . 15249622

[8] Conceicao I, Gonzalez-Duarte A, Obici L, Schmidt HH, Simoneau D, Ong ML, et al. "Red-flag" symptom clusters in transthyretin familial amyloid polyneuropathy. J Peripher Nerv Syst. 2016;21(1):5–9. doi: 10.1111/jns.12153 ; PubMed Central PMCID: PMC4788142. 26663427 PMC4788142

[9] Suhr OB, Lindqvist P, Olofsson BO, Waldenstrom A, Backman C. Myocardial hypertrophy and function are related to age at onset in familial amyloidotic polyneuropathy. Amyloid. 2006;13(3):154–9. doi: 10.1080/13506120600876849 . 17062381

[10] Soares ML, Coelho T, Sousa A, Batalov S, Conceicao I, Sales-Luis ML, et al. Susceptibility and modifier genes in Portuguese transthyretin V30M amyloid polyneuropathy: complexity in a single-gene disease. Human molecular genetics. 2005;14(4):543–53. doi: 10.1093/hmg/ddi051 . 15649951

[11] Bergstrom J, Gustavsson A, Hellman U, Sletten K, Murphy CL, Weiss DT, et al. Amyloid deposits in transthyretin-derived amyloidosis: cleaved transthyretin is associated with distinct amyloid morphology. J Pathol. 2005;206(2):224–32. doi: 10.1002/path.1759 . 15810051

[12] Ihse E, Ybo A, Suhr O, Lindqvist P, Backman C, Westermark P. Amyloid fibril composition is related to the phenotype of hereditary transthyretin V30M amyloidosis. The Journal of pathology. 2008;216(2):253–61. doi: 10.1002/path.2411 . 18729067

[13] Ericzon BG, Wilczek HE, Larsson M, Wijayatunga P, Stangou A, Pena JR, et al. Liver Transplantation for Hereditary Transthyretin Amyloidosis: After 20 Years Still the Best Therapeutic Alternative? Transplantation. 2015;99(9):1847–54. Epub 2015/08/27. doi: 10.1097/TP.0000000000000574 . 26308415

[14] Plante-Bordeneuve V, Lin H, Gollob J, Agarwal S, Betts M, Fahrbach K, et al. An indirect treatment comparison of the efficacy of patisiran and tafamidis for the treatment of hereditary transthyretin-mediated amyloidosis with polyneuropathy. Expert Opin Pharmacother. 2019;20(4):473–81. Epub 2018/11/30. doi: 10.1080/14656566.2018.1554648 . 30489166

[15] Rossi M, Baiardi S, Parchi P. Understanding Prion Strains: Evidence from Studies of the Disease Forms Affecting Humans. Viruses. 2019;11(4). Epub 2019/04/03. doi: 10.3390/v11040309 ; PubMed Central PMCID: PMC6520670. 30934971 PMC6520670

[16] Westermark GT, Fandrich M, Lundmark K, Westermark P. Noncerebral Amyloidoses: Aspects on Seeding, Cross-Seeding, and Transmission. Cold Spring Harb Perspect Med. 2018;8(1). Epub 2017/01/22. doi: 10.1101/cshperspect.a024323 ; PubMed Central PMCID: PMC5749146. 28108533 PMC5749146

[17] Westermark P. Subcutaneous adipose tissue biopsy for amyloid protein studies. Methods Mol Biol. 2012;849:363–71. Epub 2012/04/25. doi: 10.1007/978-1-61779-551-0_24 . 22528102

[18] Bergstrom J, Murphy CL, Weiss DT, Solomon A, Sletten K, Hellman U, et al. Two different types of amyloid deposits—apolipoprotein A-IV and transthyretin—in a patient with systemic amyloidosis. Lab Invest. 2004;84(8):981–8. Epub 2004/05/18. doi: 10.1038/labinvest.3700124 . 15146166

[19] Westermark GT, Ihse E, Westermark P. Development of Mouse Monoclonal Antibodies Against Human Amyloid Fibril Proteins for Diagnostic and Research Purposes. Methods Mol Biol. 2018;1779:401–14. Epub 2018/06/11. doi: 10.1007/978-1-4939-7816-8_24 . 29886546

[20] Gustafsson S, Ihse E, Henein MY, Westermark P, Lindqvist P, Suhr OB. Amyloid fibril composition as a predictor of development of cardiomyopathy after liver transplantation for hereditary transthyretin amyloidosis. Transplantation. 2012;93(10):1017–23. Epub 2012/03/08. doi: 10.1097/TP.0b013e31824b3749 . 22395298

[21] Oshima T, Kawahara S, Ueda M, Kawakami Y, Tanaka R, Okazaki T, et al. Changes in pathological and biochemical findings of systemic tissue sites in familial amyloid polyneuropathy more than 10 years after liver transplantation. Journal of neurology, neurosurgery, and psychiatry. 2014;85(7):740–6. Epub 2013/09/12. doi: 10.1136/jnnp-2013-305973 . 24023270

[22] Ihse E, Rapezzi C, Merlini G, Benson MD, Ando Y, Suhr OB, et al. Amyloid fibrils containing fragmented ATTR may be the standard fibril composition in ATTR amyloidosis. Amyloid. 2013;20(3):142–50. doi: 10.3109/13506129.2013.797890 . 23713495

[23] Ihse E, Suhr OB, Hellman U, Westermark P. Variation in amount of wild-type transthyretin in different fibril and tissue types in ATTR amyloidosis. J Mol Med (Berl). 2011;89(2):171–80. Epub 2010/11/26. doi: 10.1007/s00109-010-0695-1 ; PubMed Central PMCID: PMC3022153. 21107516 PMC3022153

[24] Marcoux J, Mangione PP, Porcari R, Degiacomi MT, Verona G, Taylor GW, et al. A novel mechano-enzymatic cleavage mechanism underlies transthyretin amyloidogenesis. EMBO Mol Med. 2015;7(10):1337–49. Epub 2015/08/20. doi: 10.15252/emmm.201505357 ; PubMed Central PMCID: PMC4604687. 26286619 PMC4604687

[25] Lai Z, Colon W, Kelly JW. The acid-mediated denaturation pathway of transthyretin yields a conformational intermediate that can self-assemble into amyloid. Biochemistry. 1996;35(20):6470–82. Epub 1996/05/21. doi: 10.1021/bi952501g 8639594

[26] Verona G, Mangione PP, Raimondi S, Giorgetti S, Faravelli G, Porcari R, et al. Inhibition of the mechano-enzymatic amyloidogenesis of transthyretin: role of ligand affinity, binding cooperativity and occupancy of the inner channel. Sci Rep. 2017;7(1):182. Epub 2017/03/17. doi: 10.1038/s41598-017-00338-x ; PubMed Central PMCID: PMC5428290. 28298647 PMC5428290

[27] Schmidt M, Wiese S, Adak V, Engler J, Agarwal S, Fritz G, et al. Cryo-EM structure of a transthyretin-derived amyloid fibril from a patient with hereditary ATTR amyloidosis. Nat Commun. 2019;10(1):5008. Epub 2019/11/05. doi: 10.1038/s41467-019-13038-z ; PubMed Central PMCID: PMC6825171. 31676763 PMC6825171

[28] Ueda M, Okada M, Mizuguchi M, Kluve-Beckerman B, Kanenawa K, Isoguchi A, et al. A cell-based high-throughput screening method to directly examine transthyretin amyloid fibril formation at neutral pH. The Journal of biological chemistry. 2019;294(29):11259–75. Epub 2019/06/07. doi: 10.1074/jbc.RA119.007851 ; PubMed Central PMCID: PMC6643022. 31167790 PMC6643022

[29] Alves-Ferreira M, Coelho T, Santos D, Sequeiros J, Alonso I, Sousa A, et al. A Trans-acting Factor May Modify Age at Onset in Familial Amyloid Polyneuropathy ATTRV30M in Portugal. Mol Neurobiol. 2018;55(5):3676–83. Epub 2017/05/21. doi: 10.1007/s12035-017-0593-4 . 28527106

[30] Santos D, Santos MJ, Alves-Ferreira M, Coelho T, Sequeiros J, Alonso I, et al. mtDNA copy number associated with age of onset in familial amyloid polyneuropathy. Journal of neurology, neurosurgery, and psychiatry. 2018;89(3):300–4. Epub 2017/10/12. doi: 10.1136/jnnp-2017-316657 . 29018163

