# Supplementary material for: Amyloid fibril composition type is consistent over time in patients with Val30Met (p.Val50Met) transthyretin amyloidosis
Source: PLoS One. 2022 Mar 31;17(3):e0266092. doi: 10.1371/journal.pone.0266092 (PMC8970372; doi:10.1371/journal.pone.0266092)
Supplement: S1 Raw images — (PDF) [file pone.0266092.s001.pdf]

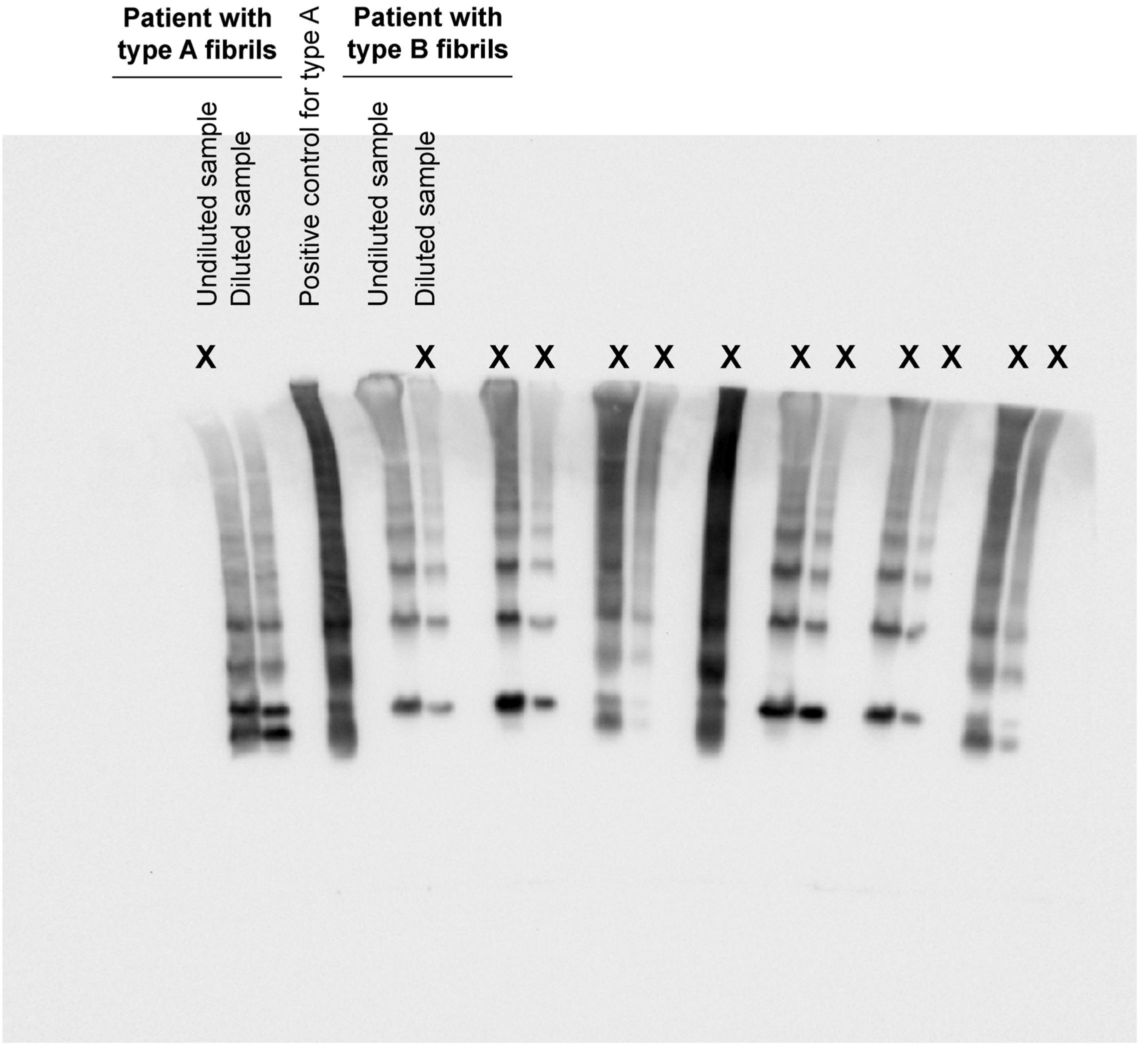

Uncropped raw western blot image underlying Fig 1.
Loaded lanes not included in Fig 1 is marked with an X.

A)

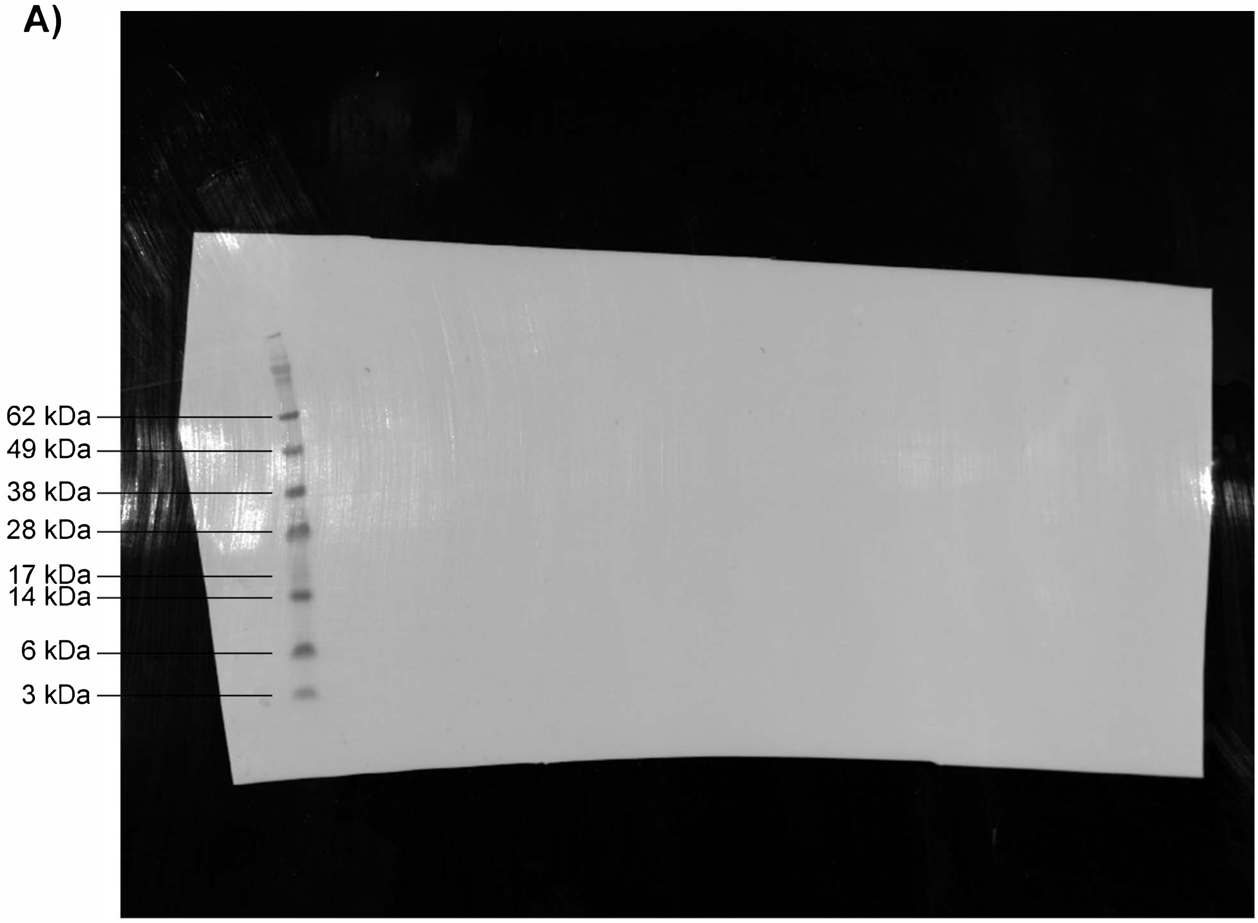

B)

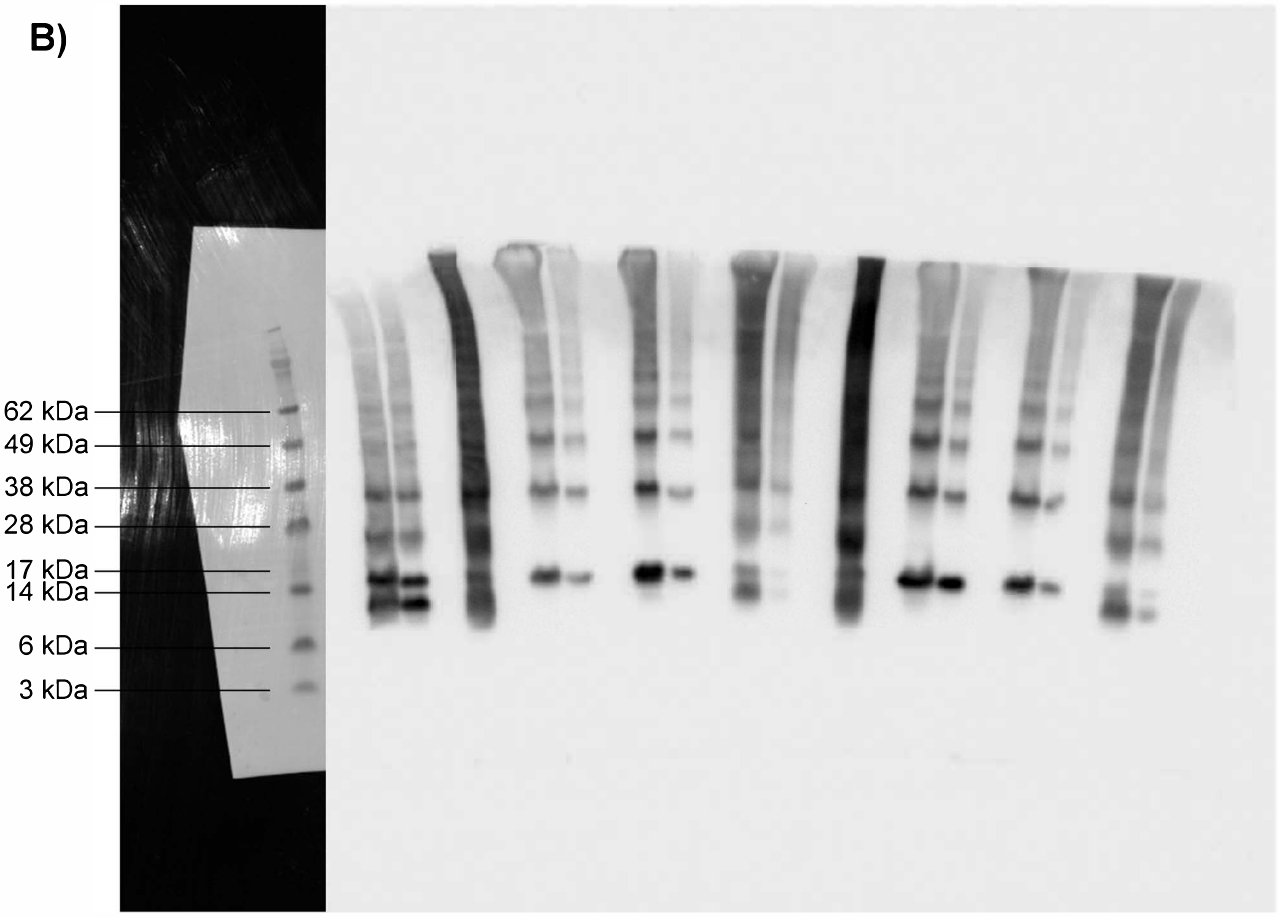

Size marker for the western blot underlying Fig 1.
A) colorimetric image of the membrane, showing the size marker,
B) the chemiluminescence and colorimetric images side by side
